# Correlation between surfactant protein B mRNA expression and neonatal respiratory distress syndrome

**DOI:** 10.3892/etm.2012.673

**Published:** 2012-08-17

**Authors:** XIAOJUAN YIN, LIHUA LI, HANXIAO FAN, WENWEN QU, LU XIE, ZHICHUN FENG

**Affiliations:** Affiliated Bayi Children’s Hospital, Beijing Military Region General Hospital, No. 5 Nan Mencang, Dongcheng District, Beijing 100700, P.R. China

**Keywords:** neonates, respiratory distress syndrome, surfactant-associated protein B, messenger RNA, deficiency

## Abstract

The aim of this study was to investigate whether surfactant-associated protein B (SP-B) mRNA deficiency is involved in the pathogenesis of neonatal respiratory distress syndrome (RDS). A total of 60 unrelated neonates who died of RDS were recruited as the RDS group and subgrouped into a ≤32-, a 32–36^+6^- and a ≥37-week group (n=20 per group) on the basis of gestational age. In addition, 60 neonates who succumbed to other diseases were enrolled as controls. The lung tissues were collected within 30 min after death. *In situ* hybridization was conducted to detect SP-B mRNA expression in the lung. The frequency of SP-B mRNA deficiency was also calculated. Among the RDS groups, the SP-B mRNA levels were significantly higher compared to those in the control group (t=7.812, P<0.001), but were comparable among RDS patients with different gestational ages (F=2.348, P>0.105). Among the control groups, the SP-B mRNA levels increased with the increase in gestational age (F=50.124, P<0.001). In the ≤32-week group, the number of cells positive for SP-B mRNA in RDS patients was markedly reduced as compared to that of the controls (t=3.185, P<0.01). In the 32–36^+6^-week group, the number of cells positive for SP-B mRNA in RDS patients was significantly smaller compared to that of the controls (t=9.342, P<0.001). In the ≥37-week group, the number of cells positive for SP-B mRNA in RDS patients was markedly smaller compared to that in the controls (t=4.238, P<0.001). Among RDS neonates, SP-B mRNA deficiency was noted in 35 patients with a frequency of 58.3%. In the control group, SP-B mRNA deficiency was noted in 8 patients with a frequency of 13.3%, which was markedly lower compared to that in the RDS group (χ^2^=26.421, P<0.001). The results of the present study therefore suggest that SP-B mRNA deficiency is involved in the pathogenesis of RDS.

## Introduction

Pulmonary surfactant is a compound comprising phospholipids and proteins. Phospholipids (mainly dipalmitoyl phosphatidyl choline, DPPC) account for 70–80% these pulmonary surfactants, while pulmonary surfactant-associated proteins including SP-A, SP-B, SP-C and SP-D account for 10% and the remaining 10% are the neutral fat (mainly cholesterol). Pulmonary surfactant is crucial in the reduction of the surface tension at the air-water interface where pulmonary surfactant is degraded and recycled. SP-B is an important pulmonary surfactant-associated protein capable of reducing or altering the surface tension by changing the surface area, preventing alveolar collapse (1–3). Studies have shown SP-B protein deficiency to be associated with the pathogenesis of neonatal respiratory distress syndrome (RDS) (4,5). The protein expression is known to be regulated by upstream genes. To explore whether the SP-B mRNA expression is associated with the pathogenesis of neonatal RDS, *in situ* hybridization was conducted to detect the SP-B mRNA expression in the lung of neonates, who were treated in the neonatal intensive care unit (NICU) between July, 2006 and October, 2010 and succumbed to RDS.

## Materials and methods

### Patients and samples

A total of 60 unrelated Han neonates in Beijing who succumbed to RDS were recruited from the NICU between July, 2006 and October, 2012 and then subdivided into ≤32-, 32–36^+6^- and ≥37-week group (n=20 per group) on the basis of gestational age. In addition, 60 neonates who succumbed to other diseases, including congenital heart diseases, bronchopulmonary dysplasia and persistent pulmonary hypertension, were enrolled as controls. The controls were also subdivided into gestational age-matched groups (P>0.05). The neonates developed RDS within 30 min to 6 h after birth and progressive dyspnea was the major clinical manifestation. Blood gas analysis showed hypercapnia and hypoxemia. These findings together with the chest X-ray results were employed to confirm the diagnosis of grade III or IV RDS. During the hospitalization, RDS patients were repeatedly treated with pulmonary phospholipids (200 mg/kg) from swine (a total of 3 or 4 times), with high frequency oscillatory mechanical ventilation being performed simultaneously. However, these patients died within 14 days. Informed consent was obtained from relatives and the whole study was approved by the Ethics Committee of General Hospital of Beijing Military Region.

### Main reagents

A DAB kit, an *in situ* hybridization kit for SP-B (Wuhan Boster Biotech Co., China), and other domestic reagents (analytically pure) were used in the present study. Digoxin-conjugated oligonucleotide probes targeting human SP-B were synthesized at the Department of Molecular Genetics as follows: i) 5′-ATGATGCCAGGTGTGTAGCC-3′; ii) 5′-AGAACCTCCCCATTGGAGC-3′; iii) 5′-GGCCTTGT GTCCAGGGAC-3′.

### Sample collection

Lung tissues were collected within 30 min after death. Samples were collected from the five lobes of RDS patients. In the control group, the lung tissues were randomly selected. The lung tissues were fixed in 4% paraformaldehyde, embedded in paraffin and consecutively cut into 5-μm sections.

### Detection of SP-B mRNA expression

*In situ* hybridization was conducted to measure the mRNA expression of SP-B in the lung according to the manufacturer’s instructions, with modification. Sections were routinely deparaffinized and dehydrated and then treated with 3% methanol in H_2_O_2_ for 20 min. After washing in distilled water three times (5 min for each), sections were treated with pepsin in 3% citric acid at 37°C for 2 min. The sections were then washed three times in PBS for *in situ* hybridization (5 min for each) and in distilled water once. Sections were fixed in 1% paraformaldehyde/0.1 MPBS (pH 7.2–7.6) at room temperature for 10 min. The sections were treated with pre-hybridization solution at 42°C for 4 h and then with digoxin-conjugated probes at 42°C for 20 h subsequent to washing three times in distilled water. After washing twice with 2X SSC at 37°C (5 min for each), 0.5X SSC at 37°C for 15 min and 0.2X SSC at 37°C for 10 min, the sections were incubated at 37°C for 30 min. Subsequently, these sections were treated with biotin-conjugated mouse anti-digoxin antibody at 37°C for 90 min. The sections were washed four times in PBS for *in situ* hybridization (5 min for each) and treated with SABC at 37°C for 30 min. The sections were then incubated with biotin-conjugated peroxidase at 37°C for 30 min after washing three times in PBS for *in situ* hybridization (5 min for each). Subsequent to washing in PBS for *in situ* hybridization four times (5 min for each), the sections were visualized with DAB for 5 min, followed by washing in water and counterstaining with hematoxylin for 1 min. The sections were treated with 1% hydrochloric acid in alcohol followed by dehydration and transparentization, and mounted. The cell nucleus was stained blue and the sections were observed under a microscope. Representative images were captured. Of note, no probe or antibody was used, in the negative control group.

### Detection of SP-B mRNA-positive cells

Positive cells had yellow brown granules in the cytoplasm following staining. Positive cells were counted at a magnification of x400. Three sections were selected from each sample and 10 fields were randomly selected from each section. A total of 30 positive cells was counted in each sample, and the number of SP-B mRNA-positive cells was determined in each subgroup.

### Determination of SP-B mRNA deficiency

The lower limit of normal of the number of SP-B mRNA positive cells was the mean - 2 standard deviations (−2SD). The sample with the number of cells lower than the lower limit of normal of the control group was defined as SP-B mRNA deficiency.

### Statistical analysis

Statistical analysis was performed with SPSS version 13.0. Data were shown as the mean ± standard deviation (±SD). The paired t-test was employed to determine comparisons between the two groups, while the one way analysis of variance was performed to determine comparisons among different groups. SP-B mRNA deficiency was analyzed with χ^2^ test. P<0.05 was considered statistically significant.

## Results

### Clinical data

RDS developed within 30 min to 12 h after birth and dyspnea progressed rapidly. Results of the blood gas analysis showed hypercapnia and hypoxemia. These findings together with the chest X-ray results confirmed the diagnosis of RDS (grade III or IV). During the hospitalization, pulmonary phospholipids from the swine were repeatedly administered at 200 mg/kg (3–4 times). High frequency oscillatory mechanical ventilation was simultaneously performed. Although comprehensive therapy was also performed, these patients died naturally or refused further treatment due to economic concern. The neonates with grade IV RDS generally died within 7 days after birth.

### SP-B mRNA expression in the lung

**Figure 1:**
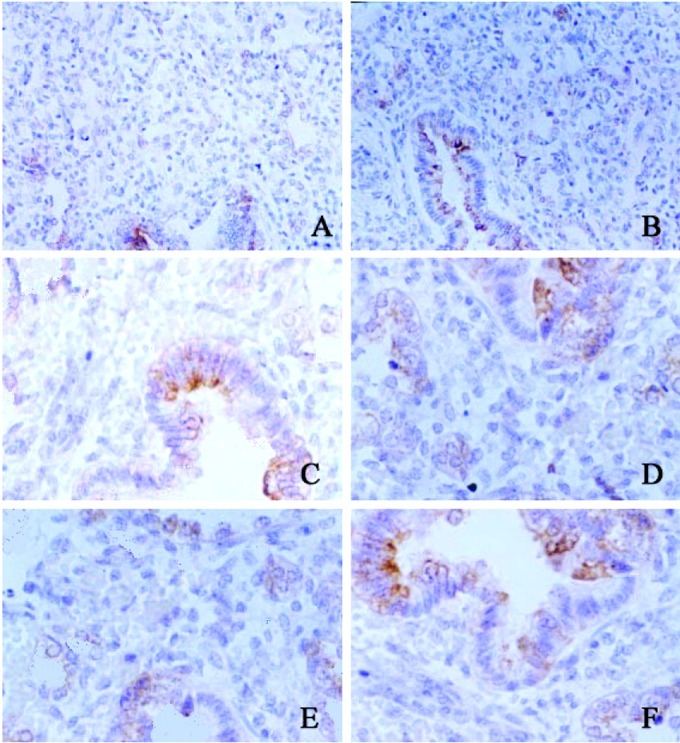
SP-B mRNA expression in different groups (positive cells, yellow). (A) RDS group (26 weeks, x100); (B) control (26 weeks, x100); (C) RDS group (34 weeks, x400); (D) control (34 weeks, x400); (E) RDS group (38 weeks, x400) and (F) control group (38 weeks, x400). RDS, respiratory distress syndrome.

The SP-B mRNA was mainly found in the cytoplasm, stained yellow. The color and the extent of staining were different, varying in the neonates in different groups, with different gestational age and with different severities of RDS. Of 60 patients with RDS, 5 had a gestational age of 24 weeks, 4 had 26 weeks, 4 had 31 weeks, 7 had 34 weeks, 5 had 36 weeks, 4 had 38 weeks and 6 had 42 weeks. The SP-B mRNA expression in the lung in the RDS neonates was markedly lower than that in the gestational age-matched controls. Four neonates with a gestational age of 38 weeks were diagnosed with RDS of grade IV and the SP-B mRNA expression was lower than that in the remaining neonates. In the control group, 5 neonates with a gestational age of 24 weeks and 3 with 26 weeks had a lower SP-B mRNA expression as compared to the lower limit of normal (Fig. 1).

### Number of SP-B mRNA-positive cells

The number of SP-B mRNA-positive cells was 34.106±15.85 in the RDS group and 53.82±11.44 in the control group, showing a significant difference (t=7.812, P<0.001). Among the RDS groups, the number of SP-B mRNA-positive cells was comparable among RDS patients with a different gestational age (F=2.348, P>0.105). However, among the controls, the number of SP-B mRNA-positive cells were found to be elevated with the increase in gestational age (F=50.124, P<0.001). In the ≤32-week group, the number of SP-B mRNA-positive cells in RDS patients was markedly reduced as compared to that of the controls (t=3.185, P<0.01). In the 32–36^+6^-week group, the SP-B mRNA-positive cells in RDS patients were significantly reduced when compared to those of the controls (t=9.342, P<0.001). In the ≥37-week group, the number of SP-B mRNA-positive cells in RDS patients was markedly lower than that in the controls (t=4.238, P<0.001) (Table I).

### SP-B mRNA deficiency

In the RDS group, there were 13 neonates in the ≤32-week group, 12 neonates in the 34–36^+6^-week group and 10 neonates in the ≥37-week group with a lower SP-B mRNA level as compared to the lower limit of normal in the control group, with a frequency of SP-B mRNA deficiency of 58.3% (35/60). In the control group, there were 5 neonates in the ≤32-week group and 3 neonates in the 34–36^+6^-week group with a lower SP-B mRNA level as compared to the lower limit of normal, with a frequency of SP-B mRNA deficiency of 13.3% (8/60). Results of the statistical analysis revealed a significant difference in the frequency of SP-B mRNA deficiency in the RDS and control groups (χ^2^=26.421, P<0.001).

## Discussion

The deficiency of pulmonary surfactant of any cause is known to potentially cause neonate RDS. Although the SP-B protein accounts for 1–2% of PS compound, SP-B is a crucial SP in the maintenance of normal surfactant function (6). SP-B has the potential to facilitate the spread of the pulmonary surfactant on the air-water interface, promote the entry of pulmonary surfactant into the interface and enhance the function of pulmonary surfactant, thereby reducing the alveolar surface tension and preventing alveolar collapse (7). Findings of recent studies have shown that SP-B reduces the alveolar surface tension and prevents alveolar collapse and also exerts an antibacterial effect (6,8). The SP-B gene located in 2p12-2p11.2, with a length of 950 bp, comprises 11 exons, while the pre-SP-B is encoded by 10 exons (9). The mature human SP-B is the product of a single gene and is encoded by exons VI and VII deriving from its precursor with a molecular weight of 42 kDa. The mature human SP-B is a lung specific protein comprising 79 amino acids with a molecular weight of 8 kDa (10). In 1981, Teja *et al* (11) identified the SP-B deficiency as an autosomal recessive disease for the first time. The SP-B deficiency has family specificity and the SP-B gene mutation varies among different races, populations and diseases. Results of another study have shown the incidence of SP-B deficiency in African Americans to be higher than in Caucasian Americans (12). The SP-B gene mutation has been shown to be associated with some respiratory diseases, including neonatal RDS, acute respiratory distress syndrome, congenital pulmonary alveolar proteinosis, adult chronic obstructive pulmonary emphysema and chronic lung diseases in children (13–15).

In the present study, the SP-B mRNA expression in the lung was detected in RDS and non-RDS neonates. Results showed that SP-B mRNA was mainly found in the cytoplasm and the SP-B mRNA level varied in different groups, including neonates with different gestational ages and those with RDS of different severities. Of 60 neonates with RDS, the SP-B mRNA expression in the lung was not enhanced with the increase in gestational age. In 5 neonates with a gestational age of 24 weeks, 4 with 26 weeks, 4 with 31 weeks, 7 with 34 weeks, 5 with 36 weeks, 4 with 38 weeks and 6 with 42 weeks, the SP-B mRNA expression was markedly lower than that in the gestational age-matched controls. In addition, four neonates with a gestational age of 38 weeks were diagnosed with RDS of grade IV, with the SP-B mRNA expression being lower than that in the remaining RDS group. Of 60 controls, 5 with a gestational age of 24 weeks and 3 with 26 weeks exhibited a lower SP-B mRNA expression than the lower limit of normal. Furthermore, the frequency of SP-B mRNA deficiency was determined to be 58.3% in the RDS group and 13.3% in the control group. The statistical analysis showed the frequency of SP-B mRNA deficiency in RDS neonates to be markedly higher than that in the controls. Thus, the SP-B protein deficiency may be attributed to the reduced transcription of SP-B mRNA, which is indirectly involved in the pathogenesis of RDS. This finding may be explained as follows: the transcription of SP-B mRNA is reduced, and then the SP-B protein expression is decreased. Thus, the formation of lamellar bodies or tubular myelin is disrupted, the mature SP-B is reduced, and the secondary mature SP-C is also decreased. Consequently, the spread and adherence of the pulmonary surfactant on the air-water interface is reduced, and the activity of pulmonary surfactant is compromised. Subsequently, the ability of the pulmonary surfactant to reduce the alveolar surface tension was compromised resulting in alveolar collapse. The fluid exudates from the capillary into the alveoli. In addition, the reduction of SP-B reduces resistance, and the antimicrobial activity of SP-B in the lung is attenuated or disappears resulting in the presence of neonate RDS. In the present study, we confirmed the hypothesis of our previous study (16). The alteration in the quality and quantity of SP-B protein suggests the changes in the upstream genes. In addition, SP-B mRNA expression was higher than the SP-B protein expression, suggesting a potential interruption in SP-B gene translation, resulting in the reduction of the SP-B protein. Therefore, SP-B in neonatal RDS should be further investigated.

Numerous studies have been conducted to investigate the correlation between SP-B deficiency and some diseases. Results have shown that SP-B deficiency is correlated with certain pulmonary diseases including neonatal RDS and chronic obstructive pulmonary (17,18). Recent ongoing studies have investigated this correlation yielding primary results (19). However, the mechanism underlying the reduction in SP-B protein should be examined. Studies on the genetic SP-B deficiency may therefore provide evidence for the clinical diagnosis and treatment of diseases in neonates.

## Figures and Tables

**Table I:** Number of cells positive for SP-B mRNA in the lung of different groups (mean ± SD).

Group	≤32-week subgroup	32–36^+6^-week subgroup	≥37-week subgroup	F	P-value
Control	41.20±9.84	58.50±3.74	61.8±6.04	50.124	<0.001
RDS	32.2±7.81	30.00±13.12	40.10±22.09	2.348	>0.105
t	3.185	9.342	4.238		
P-value	<0.01	<0.001	<0.001		
